# Effects of the Ketogenic Diet on Glycemic Control in Diabetic Patients: Meta-Analysis of Clinical Trials

**DOI:** 10.7759/cureus.10796

**Published:** 2020-10-05

**Authors:** Raghad A Alarim, Faris A Alasmre, Hammam A Alotaibi, Mohammed A Alshehri, Sara A Hussain

**Affiliations:** 1 Internal Medicine, King Khalid University, Abha, SAU; 2 Research, Prince Sultan Military Medical City, Riyadh, SAU

**Keywords:** ketogenic diet, diabetes, ldl cholesterol, glycemic control, hdl

## Abstract

Introduction

The ketogenic diet is a diet that relies on reducing carbohydrate intake to a minimum while increasing fat intake. This induces a state of ketosis where it is hypothesized to favor fat metabolism for energy instead of carbohydrates. The diet is used to treat pediatric patients with seizures to control their symptoms. Today, it is used by many to help in weight loss. Extensive research is being conducted on the benefits of the diet, as it gains popularity among patients with diabetes and obesity, to evaluate its effects on glycemic control.

Methods

This review looks at the published literature and summarizes the interventional trials that use the ketogenic diet for glycemic control. Emphasis was on pooling the results of selected variables such as weight, glycemic control, and lipid profile. The meta-analysis was conducted by a trained statistician using the Cochrane software review manager (Revman version 5.4; Cochrane, London, UK). Results were reviewed by an independent reviewer adhering to the Cochrane Collaboration's guidelines.

Results

The findings of this review show a significant effect of the ketogenic diet as compared to controls in terms of weight reduction, glycemic control, and improved lipid profile. A noticeable improvement was seen in glycated hemoglobin (HbA1c) and in high-density lipoprotein (HDL), favoring the ketogenic diet as compared to control.

Conclusion

This review concludes that the ketogenic diet is superior to controls in terms of glycemic control and lipid profile improvements, and the results are significant enough to recommend it as an adjunctive treatment for type two diabetes.

## Introduction

Type two diabetes is considered one of the major contributors to morbidity and mortality in the developed world. It is a serious public health problem with a growing prevalence, where more than 380-million patients will have the disease by 2025 [1]. Patients with type two diabetes have a lower life expectancy, higher risk of developing heart disease, diabetic neuropathy, retinopathy, and nephropathy as compared to the general population [2]. It is, therefore, a subject of extensive research to develop medications and adjunctive treatments to reduce the negative impact of the disease on patients [3]. Among the treatment options being investigated is the ketogenic diet.

The word ketogenic is a term for a low carbohydrate and high-fat diet. The idea of a ketogenic diet is to consume more calories from fat and less from carbohydrates in order to shift the basis of energy production from glucose to ketone bodies created from fat breakdown [4]. Side effects are usually mild and short in duration like headache, diarrhea, frequent urination, sweating, fatigue, hunger, anxiety and/or irritability, tachycardia, lightheadedness, and shakiness [5]. In the past, the use of the diet was for the treatment of refractory seizures in children. Today, the diet is studied for the treatment of obesity, diabetes, Alzheimer's disease, Parkinson's disease, and cancer [6].

The ketogenic diet has a noticeable effect on diabetic patients, in particular, showing decreased bodyweight, improved fasting glucose level, improved fasting insulin level, decreased cholesterol level, and diabetic medication elimination/reduction. This is hypothesized to be due to the reduction in carbohydrate intake, leading to reduced blood glucose and shifting the basic metabolism of energy from glucose to ketone bodies. This decrease in blood glucose leads to improved insulin resistance as well [7].

Many studies showed this effect of the ketogenic diet on weight loss. One such is a review that was published in the New England Journal of Medicine (NEJM) to compare the effects of a ketogenic diet with a low-fat, reduced-calorie diet. The results demonstrated that weight loss was greater with the low carbohydrate diet as compared to the low-fat diet. Although patients following either dietary method lost significant fat mass, patients who followed the ketogenic diet had more reduction in serum triglyceride levels and greater increases in high-density lipoprotein (HDL) cholesterol level as compared to the low-fat diet. This result demonstrates that the ketogenic diet can be used as an intervention for obesity and diabetes and illustrated the importance of further research into the effects of the diet [8].

For this, the objective of this review is to search, summarize, and report the effects of the ketogenic diet on glycemic control in patients with type two diabetes. A secondary objective is to assess the lipid profile on a high-fat diet and to what extent will it be elevated.

## Materials and methods

An Ovid database search was performed covering the literature published between January 2000 and July 2020. Studies were included if they met the criteria of being clinical trials, studying an adult sample of patients with type two diabetes. Studies were excluded from the meta-analysis if there was no control group. Studies with samples mixing diabetic patients with non-diabetic patients were included but the analysis was performed on the diabetic patients subgroups only. The search words included “Diabetes,” “Type 2 Diabetes,” “Ketogenic Diet,” and “Ketosis.” The included studies were evaluated individually, and the risk of bias was assessed for each study. The review followed the Cochrane handbook’s guidelines. The meta-analysis was performed using the Cochrane software Revman version 5.4 (London, UK). A methodologist was consulted to evaluate and follow the search process and perform the subsequent bias evaluation.

## Results

**Figure 1:**
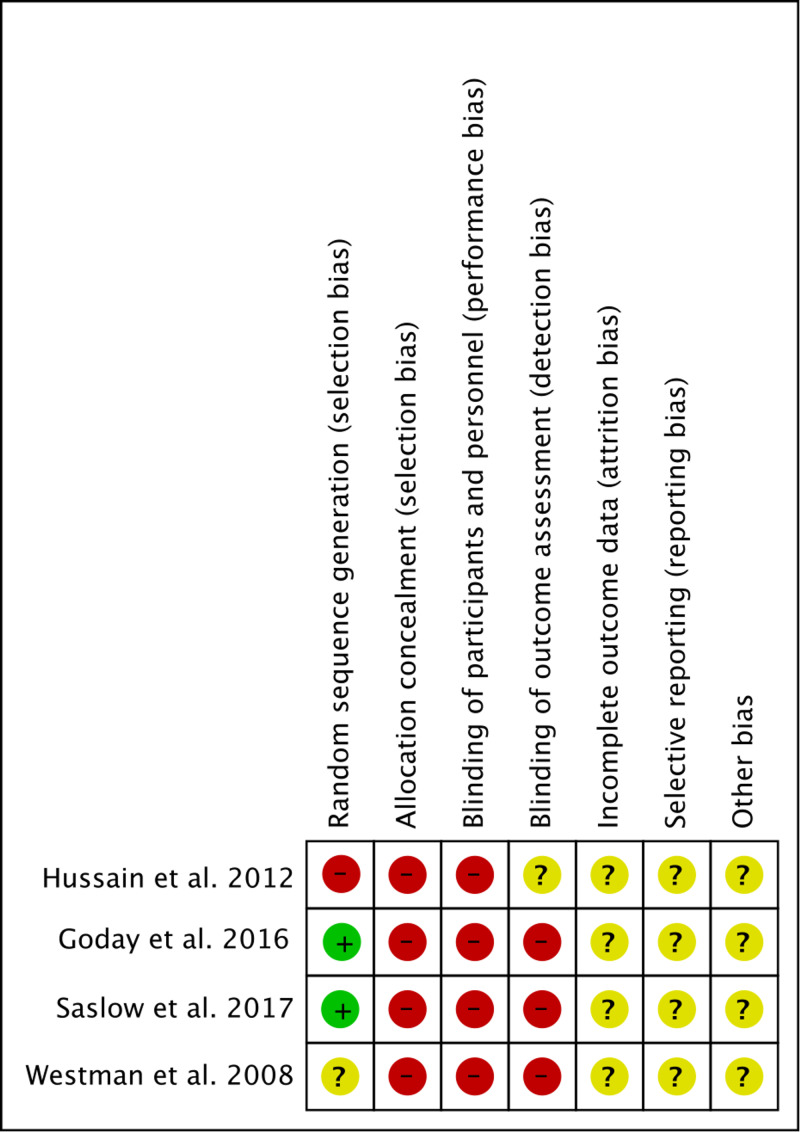
Risk of bias summary: a review of the authors' judgments about each risk of bias item for each included study Included studies [11-14]

The literature search yielded eight studies: one review, one case series, and six clinical trials, two of which are single-arm studies. Six studies were accepted for the review [9-14] but only four studies were included in the meta-analysis [11-14]. The characteristics of the included studies are presented in Table 1. The risk of bias is shown in Figure 1.

**Table 1:** Summary of included studies HbA1c: glycated hemoglobin, Kcal: kilocalorie, LCD: low carbohydrate diet, LCKD: low carbohydrate ketogenic diet, LGID: low glycemic index diet, SD: standard deviation, T2DM: type two diabetes mellitus, VLCKD: very low carbohydrate ketogenic diet, mg/dl: milligram per deciliter, mmol/l: millimole per liter, n: number of patients, p: p-value Study ID: refers to included studies [9-14]

Study ID	Design/sample	Intervention	Findings	Conclusion
Yancy et al., 2005 [9]	Single-arm 16-week interventional trial. n=28 obese diabetic patients	Patients received LCKD counseling. Target carbohydrates: <20 g/day	HbA1c decreased by 16% from 7.5 ± 1.4% to 6.3 ± 1.0% (p<0.001). Fasting serum triglycerides dropped by 42% from 2.69 ± 2.87 mmol/L to 1.57 ± 1.38 mmol/L (p=0.001)	LCKD significantly improved glycemic and lipid control. Medication discontinued in seven patients; reduced in 10.
Dashti et al., 2007 [10]	A 56-week randomized clinical trial. Sample: 64 healthy obese subject. High blood glucose n=31. Normal blood glucose n=33.	Patients received an LCKD diet. Target carbohydrates: <20 g/day. Protein: 80-100 g/day.	Fasting blood glucose level decreased significantly from 10.481 ± 3.026 mmol/L to 4.874 ± 0.556 mmol/L (p=<0.0001). Fasting serum triglycerides significantly decreased from 4.681 ± 2.468 mmol/L to 1.006 ± 0.205 mmol/L (p=<0.0001).	LCKD was very effective for improving glycemic and lipid control. Also, it helps in reducing medications in patients with type II diabetes.
Westman et al., 2008 [11]	A 24-week interventional study. Sample: 84 obese diabetic patients.	Patients received an LCKD diet. Target carbohydrates: <20 g/day (n=38). Patients received a low glycemic index diet (LGID) (n=46). Low glycemic, low calories by 500 kcal 55% of daily caloric intake from carbohydrates.	The LCKD group had a greater reduction of mean ± SD HbA1c (8.8 ± 1.8% to 7.3 ± 1.5%, p=0.009, within-group change, n=21) compared to the LGID group (8.3 ± 1.9% to 7.8 ± 2.1% p=NS, within-group change, n=29; between groups comparison p=0.03). The group that received LCKD had better results with serum triglycerides (210.4 ± 10.3 mg/dL to 142.9 ± 76.9 mg/dL) by a mean change of -67.5 as compared to the group that received LGID (167.1 ± 125.7 mg/dL to 147.8 ± 128.5 mg/dL) with a mean change of -19.3.	In the LCKD, glycemic control was greater than the LGID. Twenty of 21 (95.2%) LCKD group participants had an elimination or reduction in medication, compared with 18 of 29 (62.1%) LGID group participants (p<0.01).
Hussain et al., 2012 [12]	A 24-week diet intervention trial. Sample: 363 overweight and obese, 102 of them had diabetic patients.	Patients received LCKD and LCD and chose an LCD or LCKD counseling. Target carbohydrates: 20 g/day.	HbA1c decreased with LCKD more than LCD. Fasting serum triglycerides: decreased with LCKD more than LCD. Total cholesterol: decreased with LCKD. Blood glucose level: decreased in the two groups but LCKD had a greater effect than LCD.	LCKD had significant positive effects on serum triacylglycerol and glycemic control; there was an improvement in HbA1c.
Goday et al., 2016 [13]	A multi-centric randomized clinical trial with a duration of 4 months. Sample: 89 obese diabetic patients aged between 30 and 65 years.	Patients received LCKD and LCD. Target carbohydrates: <50 g/day.	HbA1c decreased from 6.9% to 6 % (p<0.0001) in LCKD. LCKD decreased serum triglycerides from 150.5 mg/dl to 114 mg/dl (p=0.004). Fasting glucose decreased from 136.9 mg/dl to 108,9 mg/dl (p<0.0001). Decreased oral anti-diabetic medication from 33 (73.3%) to 20 (50.0%) (p=0.0267).	LCKD is most effective in reducing body weight and improving glycemic control than a standard low-calorie diet with safety and good tolerance for T2DM patients.
Saslow et al., 2017 [14]	A 32-week randomized controlled trial. Sample: 25 obese diabetic patients, intervention group n=12, control group n=13.	Patients received VLCKD counseling. Target carbohydrates: 20-50 g/day.	HbA1c decreased in 16 weeks about -0.9% and -0.8% in 32 weeks. LCKD decreased serum triglycerides in 16 weeks about -35.5 g/dl and -60.1 g/dl in 32 weeks.	LCKD had positive effects on serum triacylglycerol and glycemic control. There was an improvement in HbA1c.

As a result of the acceptance criteria, the minimum duration of the intervention was at least 16 weeks. Only two studies specified their randomization process. None of the studies described the process of allocation concealment or blinding. This is expected in interventional trials using nutrition, where allocation concealment and blinding are not possible. In order to pool the results of accepted studies in terms of weight, body mass index (BMI), glycemic control, and lipid profile, standardized mean difference (SMD) was used with a 95% confidence interval (CI). Inverse variance weighting is used to weigh and aggregate studies.

**Figure 2:**
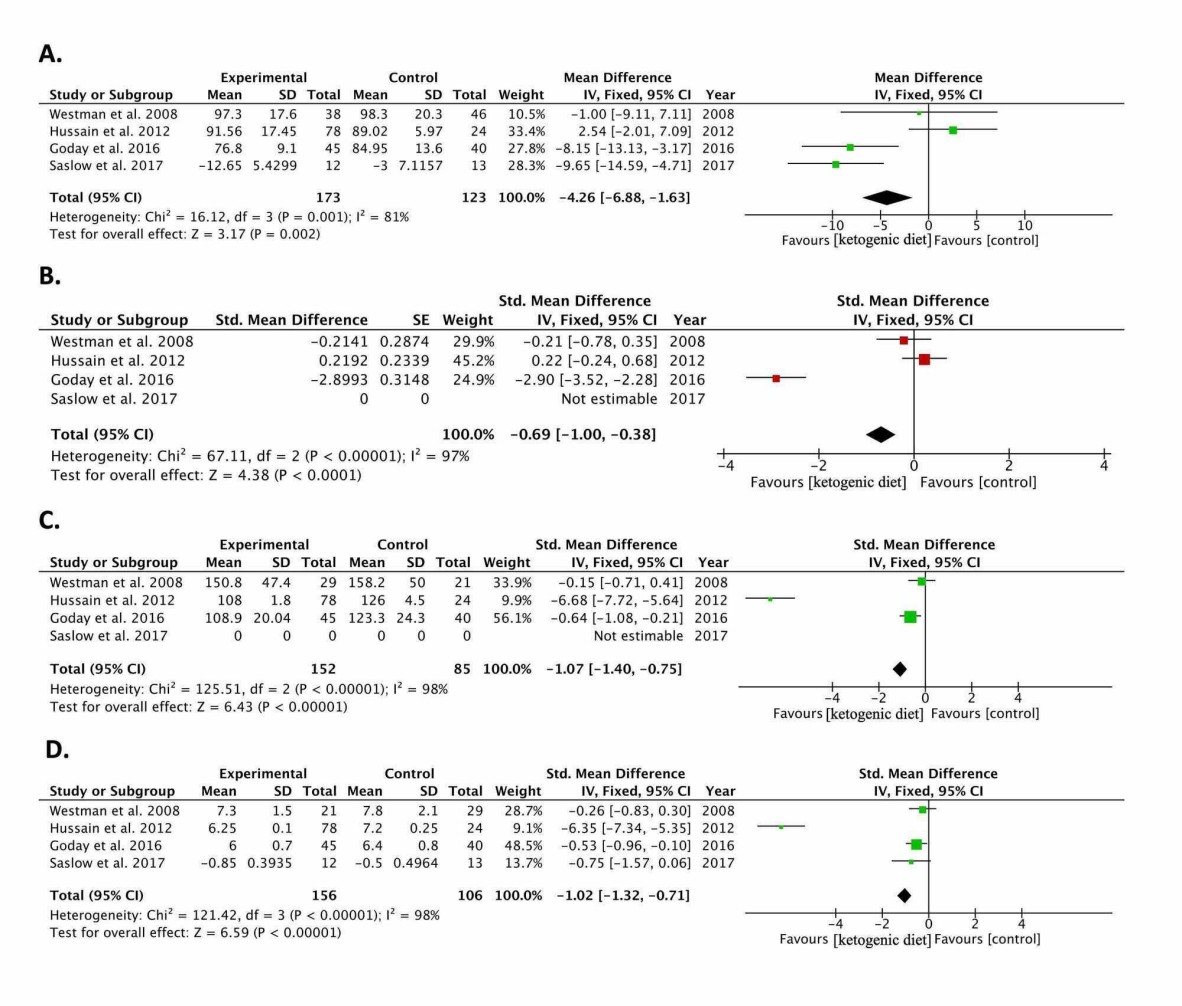
Forest plots of included randomized controlled trials comparing the effect of the ketogenic diet to controls in diabetic patients The graph shows: a. weight, b. body mass index (BMI), c. fasting blood glucose, d. glycated hemoglobin (HbA1c) Weighted standardized mean differences (95% CIs) are shown. Pooled estimates are calculated by the fixed-effect model. The squares indicate the effect of the ketogenic diet in a single study. The horizontal lines represent 95% confidence intervals (CIs). The diamond indicates the pooled effect. CI: confidence interval, IV: inverse variance Study ID: refers to included studies [11-14]

**Figure 3:**
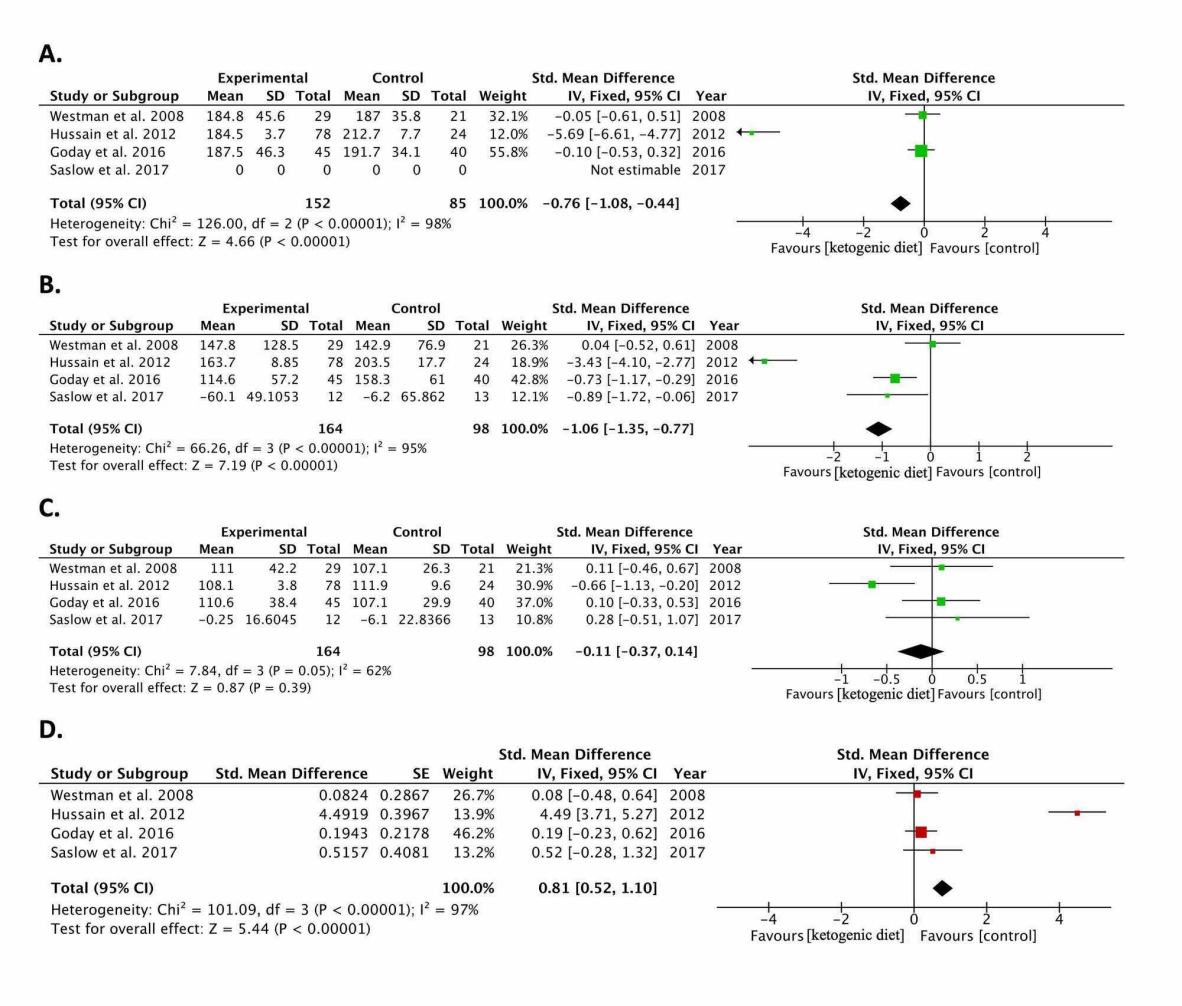
Lipid profile: Forest plots of included randomized controlled trials comparing the effect of the ketogenic diet to controls in diabetic patients The graph shows: a. cholesterol, b. triglycerides, c. low-density lipoprotein (LDL), d. high-density lipoprotein (HDL) Weighted standardized mean differences (95% CIs) are shown. Pooled estimates are calculated by the fixed-effect model. The squares indicate the effect of the ketogenic diet in a single study. The horizontal lines represent 95% confidence intervals (CIs). The diamond indicates the pooled effect. CI: confidence interval, IV: inverse variance Study ID: refers to included studies [11-14]

Results of pooling the included studies [11-14] show a significant reduction in weight (kg): -0.30 (-0.55, -0.05; p=0.02), BMI (kg/m^2^): -0.69 (-1.00, -0.38; p<0.0001), fasting blood glucose (mg/dl): -1.07 (-1.40, -0.75; p<0.00001), and glycated hemoglobin (HbA1c) (%): -1.02 (-1.32, -0.71; p<0.00001). Lipid analysis results showed a reduction in triglycerides (mg/dl): -1.06 (-1.35, -0.77) and cholesterol (mg/dl): -0.76 (-1.08, -0.44); both p<0.00001 in the ketogenic diet group as compared to controls. No statistically significant change was found between ketogenic diet and controls in terms of LDL (mg/dl): -0.11 (-0.37, 0.14; p=0.39). The pooled HDL effect size showed that the ketogenic diet raised HDL by 0.81 mg/dl (0.52, 1.10; p<0.00001) as compared to controls. The meta-analysis results of the included studies [11-14] are summarized in Figure 2 and Figure 3.

Two single-arm studies were conducted in 2005 and 2007, studying the effects of the ketogenic diet on glycemic control [9-10]. Yancy and colleagues, in 2005, conducted a 16-week single-arm diet intervention trial on 28 patients. The study looked at the relationship between low carbohydrate ketogenic diet counseling and glycemic control in overweight diabetic patients. After 16 weeks, only 21 patients completed the study. Diabetes medications were discontinued in seven patients, reduced in 10 patients, and unchanged in four patients, and HbA1c showed a significant reduction from baseline 7.5 ± 1.4% to 6.3 ± 1.0% (p<0.001). The authors concluded that adherence to a low carbohydrate ketogenic diet resulted in a significant improvement in glycemic control in obese patients with diabetes enrolled in this study [9].

In 2007, Dashti and colleagues conducted a 56-week randomized clinical trial on 64 obese subjects; 31 of them had high blood glucose and 33 had normal blood glucose. They looked at the relationship between a low carbohydrate ketogenic diet (LCKD) and BMI, body weight, glycemic control, and biochemical parameters in obese diabetic patients and in obese nondiabetics. After 56 weeks, all 64 volunteers completed the study. Fasting blood glucose level decreased significantly from 10.481 ± 3.026 mmol/L to 4.874 ± 0.556 mmol/L) P=<0.0001). Fasting serum triglycerides significantly decreased from 4.681 ± 2.468 mmol/L to 1.006 ± 0.205 mmol/L ) p=<0.0001). The authors concluded that the low carbohydrate ketogenic diet was very effective in improving glycemic and lipid control [10].

Westman and colleagues in 2008 conducted a 24-week randomized clinical trial on 84 obese diabetic patients. Volunteers were randomized to either LCKD or low glycemic index diet (LGID). The study compared the effects of LCKD and LGID on glycemic control in type two diabetes. After 24 weeks, only 48 participants completed the study. Diabetic medications were eliminated or reduced in 95.2% in LCKD group participants as compared with 62.1% in LGID group participants. The LCKD group had a greater reduction in HbA1c, better results with serum triglycerides, and greater improvement in glycemic control as compared to the LGID group [11]. In 2012, Hussain and colleagues conducted a 24-week diet interventional trial on 363 overweight and obese subjects; 102 of participants had type II diabetes. The study compared the effects of LCKD and low calories diet (LCD) in type II diabetes. All participants that enrolled in the study completed the 24-week study. The results of blood glucose level, HbA1c, fasting serum triglycerides, and total cholesterol showed a significant reduction with LCKD compared to LCD. The effectiveness of the low-carbohydrate ketogenic diet was much greater (p<0.0001) in the diabetic group on the low-carbohydrate ketogenic diet than on the low-calorie compared diet [12].

Goday and colleagues in 2016 conducted a four-month multicenter randomized clinical trial on 89 obese diabetic patients. The study compared the effects of very low carbohydrates ketogenic diet (VLCKD) versus the low-calorie diet in type two diabetes mellitus. Among the 45 patients who received VLCKD, seven discontinued the low-carbohydrate ketogenic diet before six weeks, whereas 29 completed at least the predefined maximum of 10 weeks. The effects of VLCKD showed decreased fasting glucose, HbA1c, and serum triglycerides in the LCKD group as compared to controls. The authors concluded that LCKD is most effective in reducing body weight and improving glycemic control than a standard low-calorie diet with safety and good tolerance for type two diabetes mellitus patients [13]. Saslow and colleagues in 2017 conducted a 32-week randomized controlled trial on 25 obese diabetic patients. The study looked at the relationship between VLCKD and glycemic control in obese diabetic patients. After 32 weeks, only 18 participants completed the study. Dropout was one out of 12 from the intervention group and seven out of 13 from the control group. Diabetic medications in the intervention group were reduced in one participant, increased in two participants, and unchanged in eight participants. Patients on the low carbohydrate ketogenic diet showed a significant reduction in HbA1c and serum triglycerides as compared to controls [14].

The six included studies [9-14], whether single or double-armed trials, demonstrate a clear positive trend favoring the ketogenic diet as compared to controls in every study included in this review. The pooled effect of the double-armed studies demonstrates too the effectiveness of the diet in providing glycemic control and lipid profile control in as short a time as 16 weeks.

## Discussion

The results of this meta-analysis demonstrate that the ketogenic diet is an effective tool to reduce weight, provide glycemic control, and improve the lipid profile as compared to controls. However, heterogeneity is evident in the analysis of the included variables in this review. The extent of heterogeneity is significant and, in some parameters, reached 98%. This is not uncommon in studies with small sample sizes and not uncommon as well in studies with non-uniform control like aggregating the results of slightly differing diets such as the ones included as controls in this review. Therefore, the heterogeneity of the included studies should be taken into consideration, but it is the opinion of this review that this should not influence clinical decision-making since the general results discussed in the individual studies demonstrate the superiority of the ketogenic diet as compared to the individual control comparisons irrespective of the heterogeneity in the pooled effect. The non-uniform controls could be the cause of the significant heterogeneity. Thus the results can be taken into clinical practice with no issues due to the positive trend in individual studies and concurrently in the pooled effect favoring the ketogenic diet.

It is worth noting as well that the published literature lacks studies with long-term follow-up for more than two years [15]. Longer follow-up is essential to ascertain the safety and reliability of the diet to maintain weight control, glucose control, and lipid profile control. More significantly, long-term follow-up is necessary to establish the potential harm of the diet if there is one and to what extent the said harm is present. Although there is a review of the potential therapeutic effects of the diet published in 2013, showing that it is safe in long-term use for the heart and kidneys and other systems [16], this is insufficient to recommend using the diet in the long term given the lack of long-term, large-scale prospective studies to document side-effects and demonstrate the safety profile of this diet. Thus, for the purposes of this review, the diet can safely be used, as per the latest published literature, for two years without the worry of side-effects in diabetic patients. However, a case report published in 2020 showed a serious potential side-effect of the ketogenic diet with induced fatty liver disease and elevated cholesterol and liver enzymes in a 57-year-old patient with binge eating and anxiety disorders [17]. Although this finding could be due to the patient's lifestyle prior to starting the two months trial, ruling out the ketogenic diet as a potential cause requires more studies of longer duration to ascertain the safety of the diet long term.

The findings of this review show that the effect on the lipid profile is although profound, counterintuitive given that the diet relies on fat as a source of energy. It was assumed at the beginning of this review that the lipid profile will increase due to the increase in fat consumption. However, this assumption was rejected after the analysis showed the opposite effect taking place. This result is worth further investigation to expand on the knowledge of the mechanisms leading to an elevated lipid profile in diabetic and obese patients. An explanation for the improved lipid profile is necessary and the mechanism needs revision since the status quo is that increasing fat intake has a direct effect in increasing the lipid profile, which is contrary to the findings of this review [18].

## Conclusions

The ketogenic diet showed a significant improvement in glycemic control in all studies included in this review and demonstrated a significantly improved lipid profile, including an increase in high-density lipoprotein as compared to control. Although the heterogeneity of the studies cannot be ignored, this review shows clearly that the ketogenic diet, although high in fat by definition, showed a significant and important lipid profile control coupled with weight loss and effective glycemic control. This is consistent with the effect seen in individual studies and confirmed in the aggregate effect estimate. It is, therefore, the recommendation of this review that the ketogenic diet be considered as a therapeutic intervention for diabetic patients along with medications.
